# Metabolites Produced by Fungi against Fungal Phytopathogens: Review, Implementation and Perspectives

**DOI:** 10.3390/plants11010081

**Published:** 2021-12-28

**Authors:** Sara Rodrigo, Carlos García-Latorre, Oscar Santamaria

**Affiliations:** 1 Department of Agronomy and Forest Environment Engineering, University of Extremadura, Avda, Adolfo Suárez s/n, 06007 Badajoz, Spain; saramoro@unex.es (S.R.); carloslatorre5@gmail.com (C.G.-L.); 2 Department of Construction and Agronomy, University of Salamanca, Avda, Cardenal Cisneros 34, 49029 Zamora, Spain

**Keywords:** plant diseases, fungal endophytes, metabolites, biopesticides, large-scale application

## Abstract

Many fungi, especially endophytes, have been found to produce multiple benefits in their plant hosts, with many of these benefits associated with the protection of plants against fungal diseases. This fact could be used in the development of new bio-products that could gradually reduce the need for chemical fungicides, which have been associated with multiple health and environmental problems. However, the utilization of the living organism may present several issues, such as an inconsistency in the results obtained and more complicated management and application, as fungal species are highly influenced by environmental conditions, the type of relationship with the plant host and interaction with other microorganisms. These issues could be addressed by using the bioactive compounds produced by the fungus, in cases where they were responsible for positive effects, instead of the living organism. Multiple bioactive compounds produced by fungal species, especially endophytes, with antifungal properties have been previously reported in the literature. However, despite the large amount of these metabolites and their potential, extensive in-field application on a large scale has not yet been implemented. In the present review, the main aspects explaining this limited implementation are analyzed, and the present and future perspectives for its development are discussed.

## 1. Introduction: Fungal Plant Pathogens and Control Alternatives

Considering world data from the beginning of the 21st century, microbial diseases have been estimated to cause losses of around 16% of crop yields, from which 70–80% is caused by fungal pathogens [1]. These losses are expected to increase under the new scenario created by global change, due to pests and diseases displaying more pathogenic behavior, and lower host fitness developing under the new conditions [2]. Although the traditional use of synthetic fungicides has been shown to be quite effective in fighting against fungal diseases, it presents multiple hazards. The most important among them include human health problems [3,4]; residue accumulation in multiple ecosystems, resulting in serious risks for soil organisms [5,6], aquatic organisms [7,8] and mammals [9]; and the development of fungicide resistance by pathogens [10].

Current societies are becoming increasingly aware of the drawbacks associated with the health and environmental problems derived from those traditional cropping systems. Consequently, there is a growing demand for more environmentally friendly agriculture that can reduce the use of agrochemicals. Nowadays, more than ever in the context of a global pandemic, people want healthier food, produced more respectfully with regard to the environment. These new demands are pushing the research and farming sector to look for new alternatives to fight against fungal diseases. A detailed review examining the recent alternative approaches to the use of agrochemicals to fight against plant phytopathogens was presented by El-Baky and Amara [11]. Among these new alternatives, the use of resistant cultivars may be one of the most effective methods of disease control, both economically and environmentally. However, although phytopathological aspects are a clear goal for plant breeders, there still are a very small number of resistant cultivars for a very limited number of plant pathogens. Likewise, more sustainable agricultural practices can help in avoiding fungal disease outbreaks. This is the case, for instance, for *Fusarium graminearum*, which is controlled to a great extent in wheat by using suitable crop rotation and/or inversive tillage [12]. Agricultural practices can also be applied for the control of *Phytophthora*, shown to be possible by maintaining an adequate nutritional status in citrus plants and a correct drainage system to avoid flooding [13]. Sowing cover crops acting as bio-fumigants is also an agricultural technique used against soil fungal pathogens with interesting results. Thus, Rahman et al. [14] found that mustard plants grown in intercropping with tomato, and mowed at early flowering to be incorporated in the first 15 cm of topsoil, reduced the damage from *Verticillium dahliae*.

Another very interesting alternative used to fight against fungal pathogens is biological control. Numerous organisms, parasites, predators or antagonists of pests and diseases have been reported as plant bio-protectors [15], and many of them are currently being used in the field. However, although this practice is increasingly becoming more accepted, its efficacy is highly dependent on a wide range of abiotic and biotic factors, aspects which do not yet allow for the complete replacement of agrochemicals [16]. Among biocontrol agents, in recent decades, the use of endophytic fungi has become an interesting alternative for organic agriculture. As these fungi occur inside plant tissues, in contrast with epiphytic populations which inhabit the plant surface, they have to establish a closer relationship with their hosts, which may explain the lower richness and diversity in comparison with epiphytes [17]. Although both endophytes and epiphytes have been shown to benefit plant growth and to provide plant protection [18], because epiphytic fungi are more exposed to environmental conditions, their applicability and effectiveness may be more limited. For this reason, the present review is mainly focused on (although not restricted to) fungal endophytes. An example of effective control of plant pathogens by these organisms is given by the endophyte *Induratia* spp., which has been found to produce an important diminution in the disease severity caused by three common fungal pathogens in bean crops [19]. Similarly, the symptom severity caused by *Fusarium moniliforme* in *Lolium rigidum* was reduced by the fungal endophyte *Byssochlamys spectabilis* (anamorph *Paecilomyces variotii*) when co-inoculated in plants [20]. These two examples highlight the huge potential of these microorganisms to be used as biocontrol agents, although they have also been shown to produce many other benefits in their host plants, as outlined hereafter.

## 2. Fungal Endophytes: A Promising Tool in Plant Production and Protection

Fungal endophytes are fungi living asymptomatically within plant tissues [21], both above-ground and in root tissues [22]. These fungi are extremely ubiquitous and diverse and have been found in every examined plant [23,24]. Although the role of many endophytic fungi in their host plants is not completely understood, numerous beneficial effects in their hosts have been reported in the literature. Those beneficial effects can be encompassed in two major groups: (i) those conferring adaptive advantages to the host plant and (ii) those conferring protection to the host plant against biotic or abiotic stresses. Regarding the first major group of effects, endophytes have been found to improve the nutritional status of their host plant [25,26,27,28], as well as to enhance its competitiveness against other plant species [29]. Furthermore, other fungi, such as several species from the genus *Trichoderma*, have been shown to produce growth promotion effects in their host plants [30]. Other beneficial effects found in the literature deal with increasing photosynthetic efficiency or antioxidant capacity [31,32].

Regarding the second major group of effects, while several endophytic fungi are able to confer a positive response to water stress [33,34] or to high temperatures [35] on their host plants, others have been found to provide resistance to salinity or soil heavy metals [36,37,38,39]. With regard to their protective effects against biotic factors, numerous endophytes have been described to confer resistance toward macroherbivores [40], nematodes [41,42,43], pests [44,45,46] and pathogens [47,48,49,50]. As examples of the antagonism of fungal endophytes towards fungal pathogens, *Trichoderma atroviride*, *Hypoxylon rubiginosum* and *Metarhizium anisopliae* have shown the capacity to protect their host plants against *Diplodia pinea*, *Hymenosciphus fraxineus* and *Fusarium graminearum*, respectively, among others [51,52,53]. A detailed consideration of many other examples of antagonism between fungal endophytes and fungal pathogens can be found in several reviews, such as those of Busby et al. [54], Zivanovic and Rodgers [55] and Pavithra et al. [56].

The mechanisms involved in the protective responses against fungal pathogens differ with each fungal endophyte and with the type of interaction that it establishes with its host. Although a detailed description of these control mechanisms can be found in the work of Gao et al. [57] or Busby et al. [54], they can be summarized in the following four types according to Zabalgogeazcoa [58]: (i) competition for space and nutrients, (ii) direct inhibition through antibiosis, (iii) mycoparasitism or (iv) plant resistance induction by activating the host plant defense system. In relation to the first type (competition for space and nutrients), this mechanism has been widely described in many plant–pathogen systems, such as that practiced by *Trichoderma* spp. against *Botrytis cinerea* [59]. The direct inhibition through antibiosis is mainly produced when the endophyte releases different metabolites or volatiles [60]. Regarding mycoparasitism, several genera of endophytes, such as those of *Trichoderma* spp., have been found to parasitize a wide range of fungal pathogens [61]. Multiple events are produced during mycoparasitism, such as those described by Harman et al. [62] as follows: first, the biocontrol agent has to detect other fungi and grow tropically towards them. After that and before contact, the endophyte may produce an extracellular exochitinase first, and then fungitoxic endochitinases. Once the fungi come into contact, the endophyte attaches to the host and can coil around it, forming appressoria on the host surface. Once in contact, the endophyte may produce several fungitoxic cell wall-degrading enzymes, which may result in the dissolution of the cell walls and the direct entry of the endophyte hyphae into the lumen of the target fungus.

Regarding the induction of the host plant defense system, several mechanisms can be found in the literature. For instance, the endophytic fungi *Serendipita indica* (previously known as *Piriformospora indica*) enhances resistance in barley to *Blumeria graminis* and *Fusarium culmorum* and in rice to *Thanatephorus cucumeris* (anamorph *Rhizoctonia solani*). However, in the former case, the induced plants developed structures or strategies that reduced the successful penetration of *Blumeria*, even causing local cell death [63], while in the latter case, the rice-induced plants increased their antioxidant capacity, thus limiting the severity of the pathogen [64]. *Trichoderma harzianum* has also been found to induce fungal pathogen resistance [65]. This endophyte enhances the expression of the enzymes involved in lignification in several crops, such as maize, or strengthens the epidermal cell walls in others, such as cucumber, hindering fungal pathogen penetration and colonization. All of these responses seem to be mediated by salicylic acid produced by plants as a signaling molecule. This pathway is characterized by the production of a cascade of pathogenesis-related proteins, including chitinases, glucanases and thaumatins, as well as oxidative enzymes such as peroxidases, polyphenol oxidases and lipoxygenases [62].

Among the aforementioned four types of mechanisms, it is interesting to highlight that in two of them, the fungal endophyte must be inside the plant and needs to establish some kind of interaction with its host to produce the effect. The establishment of such an interaction may require the penetration of the outermost tissues of the host by the endophyte, which may be mediated by the nature of the host and the endophyte and by the environmental conditions. For this, among other aspects, the communication between the endophyte and plant host becomes fundamental [66]. Such communication is mediated by proteins and enzymes secreted by the endophyte, which are recognized by the plant cells. As a response, plants may alter their defenses, allowing for the entrance of the fungus and the establishment of the association [67,68]. Therefore, in those cases, the protective effect is only going to be produced if multiple conditions, environmental and those related to the compatibility between organisms, are produced. In the other two types of mechanisms, the production of metabolites by endophytes is involved in the protective effect, either because they induce defense mechanisms in the host or due to their antimicrobial properties against a wide range of plant pathogens [69,70]. Thus, for a long time, fungal endophytes have been identified as an important source of novel and chemically diverse secondary metabolites [71,72,73].

## 3. Secondary Metabolites from Fungal Organisms

Fungi, especially fungal endophytes, have been identified as a prolific source of secondary metabolites with very interesting properties in a broad range of applications for different industries, such as pharmacy, the wood and food industry and agronomy [72]. Within the pharmaceutical industry, a clear example of this prolificacy has been shown by endophytic fungi isolated from marine plants, which were found to produce a great number of secondary metabolites of a diverse nature, perhaps derived from the fact that they live under extreme environmental conditions of salinity stress [74]. Thus, as an example, secondary metabolites obtained from *Fusarium equiseti* isolated from marine seaweed have shown activity against cancer cells [75]. Taritla et al. [76] also reported compounds isolated from the extracts of the marine endophyte *Aspergillus* sp. that produced cytotoxicity and apoptosis in human cancer lines. Compounds identified from the extracts of the marine endophyte *Epicoccum nigrum* isolated from seagrass from the Red Sea also showed interesting pharmacological effects, causing the growth inhibition of both Gram-positive and Gram-negative bacteria [77]. However, not only marine endophytes produce interesting secondary metabolites. In their review work, Savidov et al. [78] reported numerous fungi, several endophytes among them, that, regardless of the habitat conditions, produced biologically active terpenoids and steroids, many of them belonging to the class of highly oxygenated isoprenoid lipids (HOILs), which are important compounds involved in numerous life processes. The most important activities of these HOIL compounds reported in the literature include effects ranging from hepatoprotectant to anti-inflammatory, as well as immunosuppressant, chemopreventive or hair growth stimulant, among others [78]. Other compounds produced by fungal endophytes with further beneficial health effects, such as anticancer [79,80], anti-inflammatory, antiarthritic [81] and antiviral [82], have also been reported in the literature.

In relation to the wood and food industries, attention is being focused on various enzymes produced by endophytic fungi, such as phosphatases, esterases, lipases, proteases, cellulases, laccases, xylanases, amylases and phytases, with very interesting properties involved in multiple manufacturing processes [83,84]. In the food industry, there is also great potential for the use of other compounds, such as polyphenols, flavonoids or antioxidants, produced by endophytic fungi [85,86]. For example, several endophytic fungi, such as *Chaetomium* sp., *Curvularia* sp., *Colletotrichum* sp. and *Trichoderma* sp., isolated from *Azadirachta indica*, have been found to be an interesting source of antioxidant compounds with high free radical scavenging activity, as well as being a source of tannins and carotenoids [87], which are used in the food industry as food colorants [88] and in pharmacy due to their enhancement of the immune cell function in the body [89].

In agriculture, as active metabolites are often involved in the beneficial effects that endophytes produce on their plant hosts, they can be used to improve the general performance of crops or to protect them against biotic or abiotic stresses, in both cases increasing crop production as a consequence. A clear example of the applicability of bioactive metabolites produced by endophytes is the case of those produced by *Penicillium citrinum* isolated from *Ixeris repens*. When these metabolites were applied in *Carex kobomugi*, they produced better growth, a higher chlorophyll and carotenoid content and higher efficiency in carboxylation and in water use [90]. Another more recent example is the application of the extract produced by the ascomycete *Byssochlamys spectabilis*, formerly known as *Paecilomyces variotii*, which improved the utilization efficiency of nitrogen and phosphorus, consequently increasing the yield in rice and maize, under in-field conditions [91,92]. All of these applications evidence an extremely high diversity in the nature of the metabolites produced by fungal endophytes, many of them with antifungal properties. A complete description of the active metabolites produced by fungal endophytes and the nature of their bioactivity can be found in the review carried out by Zheng et al. [93].

## 4. Secondary Metabolites of Fungi with Antifungal Properties

**Figure 1:**
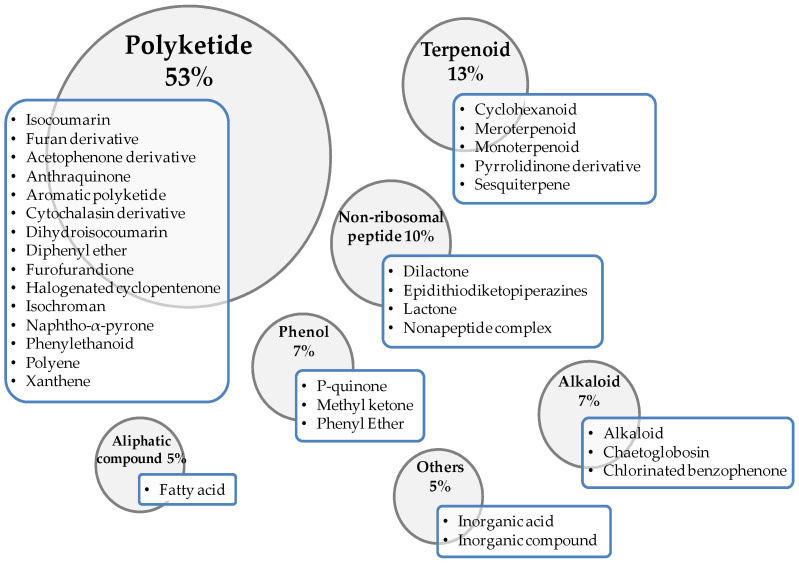
Graphical abstract of the different categories in which the fungal metabolites with antifungal properties are included, indicating the percentage of compounds in each category.

Within the wide range of secondary metabolites produced by fungi, especially fungal endophytes, many of them have antimicrobial properties, as described in detail by Mousa and Raizada [73]. Among those antimicrobial metabolites, a thorough review carried out by Xu et al. [94] showed their wide chemical diversity, with a total of 132 chemical structures being found with antifungal properties, when analyzed over the past two decades. From the compounds included in the present review, most of them (53%) are included within the polyketide compounds (Figure 1). The predominant chemical family within this category is isocoumarin, followed by furan derivative and acetophenone derivative. Twelve other chemical families are also represented within this group (Figure 1). After polyketides, the categories, ordered according to the number of metabolites with antifungal properties, are the following: terpenoids (13%), nonribosomal peptides (10%), phenols (7%), alkaloids (7%), aliphatic compounds (5%) and other compounds (5%) such as inorganic acids (Figure 1). This information is based on the most relevant substances produced by fungi showing some capacity to control any fungal phytopathogen, which are indicated in Table 1. Table 1 also displays the efficacy of the biological compounds to control the phytopathogen in relation to the corresponding chemical formulation, as well as the conditions in which the studies were performed.

Historically, one of the first examples found in the literature of a secondary metabolite produced by a fungus with antifungal properties is the case of the griseofulvin compound, which belongs to the class of organic compounds known as benzofurans. This compound, produced by *Penicillium* sp., was able to inhibit the growth of *Botrytis cinerea*, causing abnormal hyphal formations and disorientation of growth [95]. Since then, many other substances produced by fungi, especially fungal endophytes, have been described to show antifungal properties. This is the case with ergot alkaloids, produced, for instance, by *Claviceps purpurea*. These compounds, biosynthetically derived from the amino acid L-tryptophan and dimethylallyl diphosphate, have been found to exhibit, among other properties, strong antifungal activity [96]. Other alkaloids produced by fungal endophytes, such as sceptrin, a cyclobutane-containing substance, have also shown such antifungal activity [97].

From a different chemical family, some other substances with antifungal properties produced by fungi are known as acetylenic metabolites, which are isolated from *Penicillium* sp., *Aspergillus* sp. and other filamentous fungi [98,99]. These compounds, which are widespread in nature, although limited to a few groups of plants and fungi [100], are being used as the natural basis of synthetic compounds with fungicide activity [101]. Among the mechanisms of action, acetylenic compounds interfere with fatty acid production and can alter the expression of the genes that are required for fungal growth [102]. Other common chemical compounds, such as ferric chloride, potassium hydroxide and vanillin–sulfuric acid, all of them with antifungal activity, have been isolated from ethyl acetate extracts produced by the endophytic fungi *Xylaria allantoidea* [103].

**Table 1:** Metabolites produced by fungi, especially fungal endophytes, which have shown a certain control of phytopathogens.

Metabolite(s)	Producer Fungi	Fungal Pathogen(s)	Action ^1^	Conditions ^2^	Efficacy ^3^	Ref.
(12R)-12-Hydroxymonocerin	*Exserohilum* sp.	*Fusarium oxysporum*	A	IV	3%	[104]
3-(5-Oxo-2,5-dihydrofuran-3-yl) propanoic acid	*Aspergillus tubingensis*	*Fusarium graminearum*	A	IV	2-fold	[105]
4-Methylmellein, 4-hydroxymellein, 6-hydroxymellein, tyrosol	*Penicillium* sp.	*Fusarium oxysporum*	A	IV	35%	[106]
4-Prenyloxyclavatol	*Nigrospora sphaerica*	*Colletotrichum gloeosporioides*	A	IV	63%	[107]
5-Methylmellein	*Biscogniauxia mediterranea*	*Phomopsis obscurans* *Phomopsis viticola*	A	IV	5%	[108]
5-(Undeca-3′,5′,7′-trien-1′-yl) furan-2-ol	*Emericella* sp.	*Verticillium dahliae*	A	IV	49%	[109]
5-(Undeca-3′,5′,7′-trien-1′-yl) furan-2-carbonate					12%	
Bicolorin D	*Saccharicola bicolor*	*Sclerotinia sclerotiorum*	A	IV	13%	[110]
				IP-A	57%	
Brefeldin A	*Cladosporium* sp.	*Aspergillus niger*	A	IV	8-fold	[111]
Cercosporamide	*Cadophora orchidicola*	*Fusarium oxysporum* *Pestalotia diospyri* *Botrytis cinerea* *Sclerotium rolfsii* *Penicillium digitatum*	A	IV	-	[112]
Cuminic acid	*Aspergillus* spp.	*Phytophthora* spp.	A	IV	-	[113]
Epirodin	*Epicoccum nigrum*	*Brotrytis cinerea*	A	IV, IP-A	-	[114]
Ergot alkaloids	*Claviceps purpurea*	-	A	-	-	[96]
Exserolide C	*Exserohilum* sp.	*Fusarium oxysporum*	A	IV	3%	[104]
Ferric chloride, potassium hydroxide, vanillin–sulfuric acid	*Xylaria allantoidea*	-	A	IV	-	[103]
Geoxantethers A and B	Fungus from *Massarinaceae*	*Microbotryum violaceum*	A	IV	16%	[115]
Griseofulvin	*Penicillium* sp.	*Botrytis cinerea*	A	IV	-	[95]
Guignardianone C	*Phyllosticta* sp.	*Botrytis cinerea*	A	IV	52%	[96]
Hexadecanoic acid, 2,3-bis ((trimethylsilyl) oxy) propyl ester	*Trichoderma harzanium*	*Sclerotinia sclerotiorum*	A	IV	25%	[116]
Leucinostatins A and B	*Purpureocillium lilacinum*	*Phytophthora* sp.	A	IV	-	[117]
Macrosporin	*Phoma* sp.	*Fusarium graminearum*	A	IV	10%	[118]
Methyl dichloroasterrate	*Aspergillus capensis*	*Botrytis cinerea* *Monilinia fructicola* *Sclerotinia sclerotiorum* *Sclerotinia trifoliorum*	A, ISR	IV	17%	[119]
Monocerin	*Drechslera* sp.	*Botrytis cinerea* *Sclerotinia sclerotiorum*	A	IV, IP-A	-	[120]
Nigrosporamide A	*Nigrospora sphaerica*	*Colletotrichum gloeosporioides*	A	IV	10.83-fold	[107]
Not identified	*Metarhizium anisopliae*	*Fusarium graminearum* *Fusarium oxysporum*	A	IV	-	[121]
Not identified	*Metarhizium anisopliae*	*Fusarium graminearum*	A	IP-F	-	[53]
Palmitic acid, stearic acid, octadecenoic acid	*Fusarium oxysporum*	-	A, ISR, PGP	IV	-	[82]
Penicillither	*Aspergillus capensis*	*Botrytis cinerea* *Monilinia fructicola* *Sclerotinia sclerotiorum* *Sclerotinia trifoliorum*	A	IV	5%	[119]
Penochalasin K	*Penicillium chrysogenum,*	*Colletotrichum gloeosporioides*	A	IV	10-fold	[122]
		*Rhizoctonia solani*			2.66-fold	
Pestalachlorides A, B and C	*Pestalotiopsis adusta*	*Fusarium culmorum* *Gibberella zeae* *Verticillium aiboatrum*	A	IV	-	[123]
Pretrichodermamide A	*Trichoderma harzianum* *Epiccocum nigrum*	*Ustilago maydis*	A	IV	2-fold	[124]
Pseudoanguillosporin A	*Pseudoanguillospora* sp.	*Mycrobotryum violaceum*	A	IV	40%	[125]
Pyrenophorol	*Lophodermium nitens*	*Cronartium ribicola*	A	IP-G	-	[126]
Rosellichalasin	*Aspergillus capensis*	*Botrytis cinerea* *Monilinia fructicola* *Sclerotinia sclerotiorum* *Sclerotinia trifoliorum*	A	IV	32%	[119]
Speciosin U	*Saccharicola* sp.	*Cladosporium cladosporioides*	A	IV	-	[127]
Sporothriolide	*Nodulisporium* sp.	*Rhizoctonia solani* *Sclerotinia sclerotiorum*	A	IV	60%	[128]
				IP-G	79%	
Trichodermin	*Trichoderma brevicompactum*	*Rhizoctonia solani*	A	IV	1.44-fold	[129]
		*Fusarium solani*			1.21-fold	
Versicolorin B	*Aspergillus versicolor*	*Colletotrichum musae*	A	IV	-	[130]
Viriditoxin	*Byssochlamys spectabilis*	*Fusarium* spp.	A	IV; IP-G	-	[20]

^1^ Mechanism of action involved in the biocontrol: antibiosis (A); induced systemic resistance (ISR); plant growth promotion (PGP). ^2^ Conditions in which the antifungal bioactivity was obtained: in vitro (IV); in planta (or detached tissues) axenic conditions in laboratory (IP-A); in planta controlled conditions in greenhouse (IP-G); in planta uncontrolled conditions in the field (IP-F). ^3^ Efficacy: average percentage of efficacy of the biological compound in relation to the positive control (chemical fungicide) used in the corresponding study.

Furthermore, from ethyl acetate extracts (but in this case from *Trichoderma harzianum* and *Epiccocum nigrum*), pretrichodermamide A, an epidithiodiketopiperazine, has shown antimicrobial activity towards the plant pathogenic fungus *Ustilago maydis* [124]. Palmitic, stearic and octadecenoic acids, isolated from the ethyl acetate extracts of the endophyte *Fusarium oxysporum* [82], have also been identified as fungicides. Against several species of *Fusarium*, the biocontrol produced by the endophytic fungi *Byssochlamys spectabilis* when it was artificially inoculated in *Lolium rigidum* plants was attributed to the production of viriditoxin by the endophyte [20]. This molecule was identified by Wang et al. [131] as a potent bactericide due to the inhibition it causes in the formation of one of the proteins required for cell division, a mechanism that could work similarly against fungi. Leucinostatins produced by *Purpureocillium lilacinum* have displayed a broad bioactivity against bacteria and fungi. The mechanism which explains the antibiotic effect of these compounds is based on their ability to inhibit ATP synthesis in the mitochondria, as well as different phosphorylation pathways [117]. *Metarhizium anisopliae* can be considered as a promising biocontrol agent against *F. graminearum*; it produces fungistatic secondary metabolites, enhances wheat growth and elicits wheat defense responses [53]. Several other plant pathogens, such as *Alternaria solani*, *Rhizoctonia solani*, *Phytium ultimum* and *Colletotrichum lagenarium*, have shown a susceptibility to some fatty acids produced by fungal endophytes [132,133]. The important phytopathogen *Phytophthora* can be controlled by cuminic acid, a benzoic acid produced by the *Aspergillus* genus [113] and other endophytic fungi belonging to different genera [134,135,136]. Finally, *Fusarium graminearum* and *F. oxysporum* have been found to be controlled by a crude extract of the fungal endophyte *Metarhizium anisopliae* [53,121], although the active metabolite responsible for such an effect has not yet been identified. In addition to the already indicated information, the main mechanism of action responsible for the antifungal activity is related to antibiosis in most of the cases (Table 1), although this could be explained by the fact that most of the compounds were only tested in vitro. In the few cases where they were also evaluated in planta, other mechanisms, such as induced systemic resistance (ISR) in monocerin or plant growth promotion (PGP) in the compounds produced by *M. anisopliae*, might also be involved.

## 5. Current and Future Perspectives for a Large-Scale Application

Despite the vast number of studies, mostly in vitro, but also in planta, showing the antifungal properties of many fungal species, especially endophytes, which have partially been summarized in the present review, very few isolates are currently authorized by the European Union (EU) to be used against fungal phytopathogens (Table 2). Of the current few fungus-based products authorized by the EU as fungicides, none are metabolites. Therefore, the antifungal properties of many fungal species, especially endophytes, present great potential for use as biological fungicides [137], although so far they remain a research curiosity rather than a trait of commercial significance, at least with regard to agricultural purposes, as indicated by Card et al. [138] in their review on the antagonism toward plant pathogens by *Epichloë* endophytes.

The reasons explaining the very limited large-scale usage of fungal species as biocontrol agents against fungal pathogens of plants are many and can be analyzed from several points of view: efficiency on a large scale, food security, ecological impact, farmer acceptance, scaling-up of production and commercialization. Regarding efficiency on a large scale, there are numerous cases in which the promising results obtained in vitro (or even in planta under greenhouse conditions) are not translated under in-field conditions [139], where there are numerous unpredictable factors. However, the real problem lies in the fact that we do not actually know the importance of this lack of transferability, as very few studies include such in-field conditions in their research.

In-field experiments are much more complicated to carry out, as there are many uncontrolled variables that can ruin the assay, there is much more uncertainty regarding the achievement of good results and those results are usually obtained after a longer experimental time. All of these aspects, in addition to the fact that results obtained under controlled environments (either in vitro or in planta in the greenhouse) are already publishable in many scientific journals, discourage researchers from evaluating the actual applicability on a large scale of the endophytes studied. In the few cases where the biocontrol potential of fungal species was evaluated in the field, the causes for the lack of transferability, or at least for the inconsistency in the results, are related to the fact that fungi, and the effects they produce in plants, are highly influenced by environmental conditions (especially in epiphytes, but also in endophytes), the type of relationship with the plant host (which is also influenced by the environment) and the interactions with other microorganisms involved [140].

In the case of endophytic fungi (the most important ones in plant protection), the environmental conditions may be important not only to establish the association between endophyte and plant host, but also to produce the defensive response by the endophyte once the association has been established [141,142]. The variability of endophyte and host genotypes and the variability of the environmental conditions may result in a significant inconsistency in the responses observed. In some cases, even the same endophyte–host association has been found to be beneficial for the host under specific environmental conditions, while detrimental under others [143,144]. This fact may reduce the effectiveness of the association, and consequently limit its eventual application on a large scale in the field, despite the potential for endophytes to be biocontrol agents against phytopathogens. However, these above-mentioned problems are mostly derived directly from the use of the living organism. An interesting way to address such inconveniences may be the utilization of the bioactive compounds produced by the fungus (in the cases where they were responsible for a positive effect) as their efficacy may not be as dependent on environmental conditions, and the establishment of the endophyte–plant host association may not need to be produced. This fact can be observed in the efficacy of the metabolite sporothriolid (in Table 1), which was relatively similar under both in vitro and greenhouse conditions. Nevertheless, as in the case of the living organism, metabolites have also been little studied to date under in-field conditions (Table 1). Therefore, further efforts should be made to evaluate the transferability of the positive results found under laboratory conditions to the field.

With respect to food security and ecological impact, it may be important to evaluate if the application of the fungal species produces harmful effects in humans, livestock, wildlife, flora, mycorrhizal fungi, invertebrates, beneficial microorganisms or ground water. This is a vital issue as many substances that are produced by fungi have been found to present toxic effects that could affect non-target organisms, such as insects [46], plants [145] or mammals [146]. Therefore, although a fungus may exhibit strong antifungal activity, its utilization as a biocontrol agent should be avoided or limited if toxicity for other organisms is detected. Furthermore, toxicity should be evaluated under different conditions and hosts because, when a living organism is used, several other substances could be produced by the fungus in addition to those causing the antifungal activity, as the production of multiple metabolite types is quite common in many fungal species, especially endophytes [110,147]. In the case of directly using the metabolite instead of the living organism, this multiple production may not be produced, thus facilitating the evaluation process. In any case, strict regulations in different countries (such as those in the EU) for the utilization of active substances as fungicides, which undoubtedly serve to protect our health, have also somewhat limited further development of this type of product.

Another reason for the scarce utilization of this type of product in the field is the still low acceptance by farmers, who are still quite skeptical about the efficiency of its application in comparison with chemical pesticides. This founded perception is based on the fact that most of the fungi-based products are able to reduce the incidence of disease, but they do not produce a complete suppression of the pathogen. In very few cases, the efficiency of the biological product is superior to the chemical fungicide. Only when using the metabolite instead of the living organism, such as in some synthetic derivatives from griseofulvin, a better efficiency has been found in comparison with chemical fungicides, such as hymexazol, thiophanate-methyl, ketoconazole or bifonazole, for the control of several phytopathogens [148,149]. Nevertheless, the cases in which the biological compound has been found to be more efficient than the corresponding chemical substance are mainly limited to in vitro experiments (Table 1). Further experiments in the field should be conducted to evaluate if biological control can be maintained under such uncontrolled conditions. In any case, most of the cases present an efficacy percentage below 50% (Table 1). Therefore, while these new fungi-based products do not present at least the same efficacy as that of the chemical options, farmers may be unwilling to use these types of substances, unless consumers are able to pay a higher price for foods produced under such conditions. Likewise, the management and application processes of a living organism are usually more complicated than those of a chemical compound, due to the storage requirements, the application medium preparation and the application procedures, which may be onerous for farmers. This fact may aggravate the already low motivation of farmers to use these types of products. Once again, the use of metabolites instead of living organisms may facilitate such utilization, as the procedure for managing and applying the product may be similar to that of the chemical substances. Furthermore, as explained earlier, the metabolite effect may not be as dependent on external conditions, so its efficiency may be higher than that of the living organism.

Finally, commercializing companies are also very important actors in the large-scale implementation of this type of product, as they are responsible for the development, production and commercialization of the product. Therefore, the scarce utilization of fungi-derived products so far may be partially because many conventional fungicide-manufacturing companies could be unwilling to adapt to the different procedures that this form of manufacturing requires. A major investment is required for such an adaptation in a sector perhaps not mature enough yet, as the farmers’ unwillingness to use these products may indicate. Nevertheless, several studies, such as that of Ganeshan et al. [150], have tried to help the industry translate the research from a laboratory scale to a large-scale commercial production manufacturing process by including the main steps for the optimization processes in bioreactors. However, more important than the production process may be the initial development of the active substance to be further produced and commercialized. This development could take a very long time, requiring strong investment and, most importantly, very sophisticated know-how, along with a technological and fully researched background. All of these important aspects discourage many companies from undertaking the long and winding road to the development of a new product. A suitable option for companies interested in the production of these products may be the establishment of consortia with universities or scientific centers that can manage and take the lead with regard to the most technological tasks and the research. In several countries, such as Spain, the most recent governmental policies encouraged these kinds of associations [151] in order to favor small or medium-sized companies that might be unable to accomplish such a development on their own.

## 6. Concluding Remarks

Despite the huge potential for using fungal species, especially endophytes or their active metabolites, in the control of fungal diseases once a commercial bio-pesticide is developed, the interest in their application is relatively recent, coinciding with increasing societal concern about environmental issues and their relationship with human health. For this reason, the literature explaining the techniques for industrializing the production of endophytes or their metabolites is still scarce, especially for a large-scale application of metabolites. One of the few examples can be found in the work conducted by Wang et al. [152], where a procedure for industrializing the production of metabolites from *Paecilomyces variotii* was established. In general, the development of a commercial bio-fungicide based on the metabolites produced by fungal species could involve the following stages: (i) isolation and identification of novel fungal species, especially endophytes, from sources that could favor further positive effects; (ii) screening and selecting fungi that could potentially develop the target effect (in our case antifungal activity); (iii) evaluation of artificial inoculation of the selected fungal species in target plant hosts; (iv) production of extracts under various conditions; (v) isolation and identification of the metabolites presented in the extracts; (vi) evaluation of the bioactivity of each isolated metabolite; (vii) optimization of the fermentation conditions to maximize the production of the active metabolite; (viii) development of the commercial formulations by evaluating the best ingredients; (ix) evaluation of the efficiency of the different formulations in the field; and (x) establishment of a marketing strategy and commercialization.

Most of the research studies have been focused on one or several of the points from (i) to (vi). However, the little effort made so far by researchers and companies to develop the points from (vii) to (x) may explain the minimal relevance that this type of product currently has. One of the few works, in this case involving point (vii) (optimization of the fermentation conditions), is the aforementioned study carried out by Ganeshan et al. [150], where a review of the concepts, challenges and perspectives regarding the scale-up production of plant endophytes in bioreactors is given. Therefore, these points should be strengthened in order to successfully implement these bio-products in agriculture. In our opinion, the cooperation between research institutes and companies could be of key importance for the achievement of this goal, which undoubtedly could also be favored by public policies.

## Figures and Tables

**Table 2:** Fungal species authorized by the European Union to be applied against fungal pathogens.

Fungal Isolate	Applied Crop	Target Fungal Pathogen	Application Form
*Ampelomyces quisqualis*	Grapes, tomato, pepper, aubergine	Powdery mildew	Spores
*Aureobasidium pullulans*	Pome fruit	*Erwinia amylovora*	CFU
*Candida oleophila*	Apples/pears	Postharvest diseases	CFU
*Clonostachys rosea*	Fruiting and leaf vegetables, seedlings, ornamentals, pot plants, cut flowers, wheat, corn, onion, potato, leek, berries	Seed borne and soil borne fungi, such as *Fusarium*, *Pythium*, *Rhizoctonia* and *Phytophtora*. Foliar pathogens, e.g., *Botrytis* and *Didymella*	CFU
*Coniothyrium minitans*	Winter rape, lettuce, cucumber, beans, sunflower	*Sclerotinia sclerotiorum*, *S. minor*	Spores
*Pythium oligandrum*	Oilseed rape	*Sclerotinia sclerotiorum*, *Leptoshaeria maculans*	Oospores
*Saccharomyces cerevisiae*	Pome fruits (apple, pear, quince, medlar, nashi)	*Monilinia*, *Botrytis*, *Alternaria*	CFU
*Trichoderma asperellum*	Tomato, pepper, cucumber, courgette, carnation plants growing in the greenhouse	Soil pathogens: *Pythium* spp., *Rhizoctonia* spp., *Phytophthora* spp., *Phoma* spp., *Verticillium* spp. and *Fusarium* spp. *Fusarium oxysporum*	CFU
*Trichoderma atroviride*	Grapevine, tomato	Wood decay diseases. *Pythium* spp. *Rhizoctonia* spp. *Fusarium* spp.	CFU
*Trichoderma gamsii*	Tomato, pepper, cucumber, courgette	*Phythophthora* sp. *Fusarium* sp. *Rhizoctonia solani* *Pythium* sp. *Sclerotinia sclerotiorum*	CFU
*Verticillium albo-atrum*	Elm trees	Vascular fungus: *Ophiostoma novo*-*ulmi*	Spores

(https://ec.europa.eu/food/plant/pesticides/eu-pesticides-database, accessed on 11 November 2021).

## Data Availability

All data are included in the present study.
